# Screening and characterization of aptamers recognizing the periodontal pathogen *Tannerella forsythia*

**DOI:** 10.1002/2211-5463.13772

**Published:** 2024-02-02

**Authors:** Danuta Mizgalska, Stanisław Malicki, Anna Golda, Barbara Chruścicka‐Smaga, Jan Potempa

**Affiliations:** ^1^ Department of Microbiology, Faculty of Biochemistry, Biophysics and Biotechnology Jagiellonian University Kraków Poland; ^2^ Laboratory of Proteolysis and Post‐translational Modification of Proteins, Malopolska Centre of Biotechnology Jagiellonian University Kraków Poland; ^3^ Department of Oral Immunology and Infectious Diseases University of Louisville School of Dentistry KY USA

**Keywords:** aptamers, mirolysin, periodontal disease, periodontal pathogen, periodontitis, *Tannerella forsythia*

## Abstract

Periodontal disease is one of the most common forms of inflammation. It is currently diagnosed by observing symptoms such as gingival bleeding and attachment loss. However, the detection of biomarkers that precede such symptoms would allow earlier diagnosis and prevention. Aptamers are short oligonucleotides or peptides that fold into three‐dimensional conformations conferring the ability to bind molecular targets with high affinity and specificity. Here we report the selection of aptamers that bind specifically to the bacterium *Tannerella forsythia*, a pathogen frequently associated with periodontal disease. Two aptamers with the highest affinity were examined in more detail, revealing that their binding is probably dependent on mirolysin, a surface‐associated protease secreted by the *T. forsythia* type‐9 secretion system. The aptamers showed minimal cross‐reactivity to other periodontopathogens and are therefore promising leads for the development of new tools to study the composition of the periodontitis‐associated dysbiotic bacteriome as well as inexpensive new diagnostic assays.

## Graphical abstract

In this study, we identified aptamers with high specificity for the periodontal pathogen *Tannerella forsythia*, commonly linked to periodontal disease. Two top‐affinity aptamers were further investigated, demonstrating minimal cross‐reactivity with other periodontopathogens. These aptamers hold potential for developing novel tools to explore the composition of the dysbiotic bacteriome in periodontitis and cost‐effective diagnostic assays.

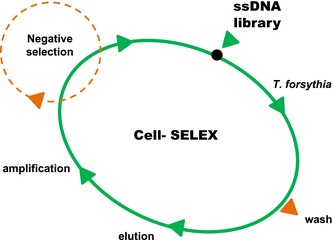

**Abbreviations**
BSA: bovine serum albumin
D‐PBS: Dulbecco's phosphate‐buffered saline
*K*_d_
: dissociation equilibrium
MFI: mean fluorescence intensity
NAM: *N*‐acetylmuramyl acid
OD_600_
: optical density at 600 nm
SELEX: selective expansion of ligands by exponential enrichment
ssDNA: single‐stranded DNA
T9SS: type‐9 secretory system

Aptamers are short, single‐stranded nucleic acids or peptides that fold into three‐dimensional structures, allowing them to bind specific targets with high affinity [1]. Nucleic acid aptamers are obtained from a random library of synthetic oligonucleotides by multiple rounds of screening and affinity‐based selection, a process known as selective expansion of ligands by exponential enrichment (SELEX), ultimately yielding a small group of aptamers with very high affinity for the target [2]. Aptamers can bind diverse targets, ranging from simple ions to small molecules, complex molecules such as proteins, and even whole viruses and cells [3].

Aptamers can be used in the same way as antibodies for diagnostic and therapeutic applications, but are less expensive to produce because aptamer synthesis, screening, and selection are carried out entirely *in vitro* [3]. They can be chemically modified to increase their stability, affinity, and bioavailability, or can be prepared from synthetic nucleic acid analogs known as xeno nucleic acid [4]. Aptamers also have low immunogenicity and can cross tissue barriers. Despite these advantages, the therapeutic use of aptamers has been limited [5]. Only one aptamer‐based drug has been approved by the US Food and Drug Administration, an RNA aptamer known as pegaptanib that targets vascular endothelial growth factor to treat neovascular age‐related macular degeneration [3]. In contrast, aptamers have been widely explored as diagnostics, especially in cancer and infectious diseases as well as food safety testing and environmental monitoring [5]. Diagnostic biosensors include a biorecognition element that specifically binds to a target and a transduction mechanism that detects such binding events [6]. In aptasensors, the recognition molecule is a selected aptamer, and the most common detection mechanisms are based on optical or electrochemical signal transduction. Such configurations can be used for example in point‐of‐care diagnostic systems [7, 8].

Periodontal disease affects gingival and periodontal tissues and is one of the most common inflammatory diseases in the world. The severity of periodontal disease symptoms graduates from healthy gingiva, through gingivitis to various stages of periodontitis. The disease classification is based on several distinct clinical presentations, with different ages of onset and rates of progression [9]. During its final stages, periodontitis triggers alveolar bone resorption with loss of dentition and is associated with other systemic diseases such as Alzheimer's disease and rheumatoid arthritis [10]. The diagnosis and monitoring of periodontal disease progression and treatment are currently based on the observation of bleeding on probing, gingival attachment loss, and periodontal pocket depth. However, the use of salivary biomarkers such as interleukin‐1β and matrix metalloproteinase 8 has also been proposed [11, 12]. This is potentially advantageous because it would allow earlier diagnosis and the implementation of preventative measures before symptoms become severe.

The dysbiotic bacterial community on the tooth surface is regarded as the main etiological factor associated with periodontitis and could therefore provide a series of biomarkers for diagnostic testing. Combined with genetic and lifestyle factors, the dysbiotic microbiome deregulates immune responses, leading to chronic inflammation and the direct erosion of tooth‐supporting tissues. Dysbiosis involves gradual changes in the microbial community, resulting in the predominance of periodontitis‐associated anaerobes including the red complex bacteria (*Porphyromonas gingivalis*, *Tannerella forsythia*, and *Treponema denticola*) as well as emerging pathogens such as *Filifactor alocis* and *Fretibacterium fastidiosum* [13, 14].

*Tannerella forsythia* is a Gram‐negative, anaerobic, asaccharolytic bacterium from the phylum Bacteroidetes. It is characterized by slow, fastidious growth *in vitro* and a dependence on externally provided *N*‐acetylmuramyl acid (NAM). *T. forsythia* cells are covered by a protective semi‐crystalline S‐layer composed of two glycoproteins that increase its virulence. The S‐layer components, as well as proteases that promote disease progression, are secreted by a type‐9 secretory system (T9SS) [15, 16]. Clinically, *T. forsythia* is positively correlated with the severity of periodontal disease, with higher bacterial loads increasing the mean attachment loss in a longitudinal study [17, 18]. The detection of *T. forsythia* cells or associated biomarkers could therefore provide a useful diagnostic assay that could improve the outcome for periodontitis patients by allowing earlier intervention and treatment evaluation.

Here we set out to isolate aptamers that specifically detect *T. forsythia* as the basis of a future research tool to investigate the oral microbiome in periodontitis patients and potentially also for the development of new diagnostic assays. We used the SELEX procedure to isolate two high‐affinity aptamers and tested a range of *T. forsythia* mutants to identify the corresponding molecular targets.

## Materials and methods

### Bacterial cultures

*Tannerella forsythia* strain ATCC 43037 and its mutants were cultivated in 30 g·L^−1^ tryptic soy broth containing 5 g·L^−1^ yeast extract, 0.5 mg·L^−1^ hemin, 1 mg·L^−1^ menadione and 10 mg·L^−1^ NAM. For the isolation of single colonies, we supplemented the medium with 5% sheep blood and solidified it with 1.5% agar. The cultures were incubated at 37 °C in an anaerobic chamber (Whitley A85 Workstation; Don Whitley Scientific, Bingley, UK). Similarly, *Tannerella serpentiformis* (HOT‐286) was cultured; however, in the presence of a helper species *Cutibacterium acnes*. *P. gingivalis* W83, *Porphyromonas gulae* ATCC 51700, *Prevotella intermedia* 17, *Aggregatibacter actinomycetemcomitans* ATCC 33384, *Fusobacterium nucleatum* ATCC 10953, *Streptococcus oralis* ATCC 35037, *Streptococcus sanguinis* ATCC 10556, *Streptococcus gordonii* ATCC 10558, and *Parvomonas micra* ATCC 33270 were grown as described above, with NAM omitted from the medium. *Escherichia coli* DH5α cells were cultivated in 20 g·L^−1^ lysogeny broth or on plates of the same medium solidified with 1.5% agar.

### Mutagenesis of *T. forsythia*

The *T. forsythia* mutant deficient for the S‐layer was obtained by homologous recombination as previously described [19]. Briefly, vector pTfsAB_LONG_cat_pUC57 (containing a chloramphenicol resistance cassette flanked by an 845‐bp sequence upstream of the *TfsA* start codon and a 1274‐bp sequence downstream of the *TfsB* stop codon) was synthesized by GenScript Biotech, Piscataway, NJ, USA. The vector was introduced into *T. forsythia* by electroporation, and recombinant clones were selected on medium supplemented with 10 μg·mL^−1^ chloramphenicol. Following the isolation of genomic DNA using a Genomic Mini isolation kit (AA Biotechnology, Krakow, Poland), the presence of the insert was verified by PCR in a 40‐μL reaction comprising 30 ng genomic DNA, 0.8 U Phusion polymerase (Thermo Fisher Scientific, Waltham, MA, USA), 1× HF buffer, 0.37 mm dNTPs (Thermo Fisher Scientific), and 0.045 mm of each primer (for the upstream fragment, forward primer seqDelTfsAUP_R 5′‐GAT CAT GCG TTG CGC GTG AT‐3′ and reverse primer seqcat_F 5′‐CGG TCT GGT TAT AGG TAC ATT G‐3′; for the downstream fragment, forward primer seqDelTfsAB2DW_R 5′‐ATG TCA GGC TTT CAG CCC TC‐3′ and reverse primer seqcat_F 5′‐GTC TGT GAT GGC TTC CAT GTC‐3′). The PCR program began with initial denaturation for 2 min at 98 °C, followed by 30 cycles of denaturation (98 °C, 10 s), primer annealing (63 °C, 20 s), and elongation (72 °C, 30 s), and a final elongation step (72 °C, 5 min). The reaction products were resolved by 1% agarose gel electrophoresis and purified using the GeneJET extraction kit (Thermo Fisher Scientific) before sequencing. A positive clone was also confirmed by SDS/PAGE followed by staining with Coomassie Brilliant Blue as previously described [20].

### Cell‐SELEX

The aptamers were selected using a cell‐SELEX method [21]. The SELEX library consisted of 80‐nt single‐stranded DNA (ssDNA) molecules with a 40‐nt variable central region flanked by 20‐nt primer sequences (5′‐CAT GCT TCC CCA GGG AGA TG‐N40‐GAG GAA CAT GCG TCG CAA AC‐3′). The library was synthesized at the 0.2‐μm scale and was purified by HPLC (IBA, Gottingen, Germany). Before each selection cycle, the ssDNA molecules were denatured for 5 min at 92 °C followed by slow renaturation (10 min at 4 °C and 15 min at room temperature). *T. forsythia* growing on solid medium was harvested in 1 mL (Dulbecco's phosphate‐buffered saline (D‐PBS; Thermo Fisher Scientific)) supplemented with 1% bovine serum albumin (BSA; Bioshop, Burlington, ON, Canada). The cells were centrifuged (5000 ***g***, 5 min, room temperature) and suspended in the same buffer before measuring the optical density at 600 nm (OD_600_). We then took 10^8^ cells for the initial round of selection in binding buffer (PBS containing 5 mm MgCl_2_, 10 mm CaCl_2_, and 0.01% Tween‐20) supplemented with 40–120 μg·mL^−1^ yeast tRNA (Thermo Fisher Scientific) and 125 μg·mL^−1^ BSA. Binding was promoted by incubating the aptamers with the bacteria for 1 h at room temperature with constant shaking in 100 μL nuclease‐free water (Sigma‐Aldrich, Saint Louis, MO, USA). The cells were washed twice with 1% BSA in D‐PBS followed by thermo‐elution at 95 °C for 10 min, followed by cooling to 4 °C for 10 min. Cell remnants were separated by centrifugation (15 000 ***g***, 10 min, room temperature). The eluted aptamer pool was then amplified by PCR in an 850‐μL reaction containing 100 μL of the eluted ssDNA pool, 0.6 μm of each primer (fluorescence‐labeled FAM_APT_F 5′‐CAT GCT TCC CCA GGG AGA TG‐3′ and biotinylated APT_R 5′‐GTT TGC GAC GCA TGT TCC TC‐3′), 0.2 mm dNTPs, 3 mm MgCl_2_, and 10 U Taq DNA polymerase in 1× buffer (Thermo Fisher Scientific). The PCR program comprised 30 cycles of denaturation at 95 °C for 2 min, annealing at 53 °C for 30 s, and extension at 72 °C for 30 s, followed by a final extension step at 72 °C for 5 min. The PCR products were purified using a MinElute kit (Qiagen, Venlo, the Netherlands) followed by ssDNA separation on streptavidin‐coated magnetic beads (New England Biolabs, Ipswich, MA, USA). The 30 μL of 0.2 m NaOH used for elution was neutralized with 15 μL 0.3 m HCl and buffered with 45 μL of 2× PBS. The ssDNA samples were stored at −20 °C for the next selection cycle, of which there were eight in total. Selection was initiated with 2 nmol of the initial library and 10^8^ cells. From the second round, the selective pressure was strengthened by reducing the concentration of the ssDNA aptamers (to 40 pmol from cycle 3) and bacterial cells (gradual decrease to 3 × 10^7^ cells in the final cycle). Moreover, two contra‐selections were performed against *E. coli* cells (10^7^ cells, cycle 6) and *P. gingivalis* (2 × 10^7^ cells, cycle 7). The aptamer pool after eight selection rounds was prepared for Illumina next‐generation sequencing (Genomed, Warszawa, Poland).

### Flow cytometry

To evaluate the binding of aptamers to bacterial cells, we used the 5′‐FAM‐labeled aptamers TF1 and TF2 as well as the nonspecific aptamer KO‐1. The cells were harvested in 1% BSA in D‐PBS, washed once, and resuspended. We then combined 100 μL of washed bacteria (6.8 × 10^7^ cells) with different concentrations of labeled aptamers and incubated them for 1 h at room temperature in the dark. The cells were washed three times with 1% BSA in D‐PBS and resuspended in 400 μL of the same buffer. After incubation, cells were analyzed by flow cytometry using a FACSCalibur or LSR Fortessa device (BD Biosciences, Franklin Lakes, NJ, USA). Graphs were prepared using flowjo v10.6.2 (FlowJo, Ashland, OR, USA). The dissociation equilibrium (*K*_d_) was obtained by matching the dependence of the mean specific binding fluorescence intensity to the aptamer concentration (0–250 nm).

### Bioinformatic analysis

Aptamer pool sequencing results were analyzed as previously described [22]. Shortly, flanking region‐trimmed unique sequences were aligned and visualized as a phylogram of the 10 most abundant aptamers by the clustal omega program (https://www.ebi.ac.uk/Tools/msa/clustalo/). Aptamer secondary structure prediction was performed with the RNAstructureWeb platform (https://rna.urmc.rochester.edu/RNAstructureWeb/index.html).

## Results

### Selection and identification of aptamers binding to *T. forsythia* cells

**Fig. 1:**
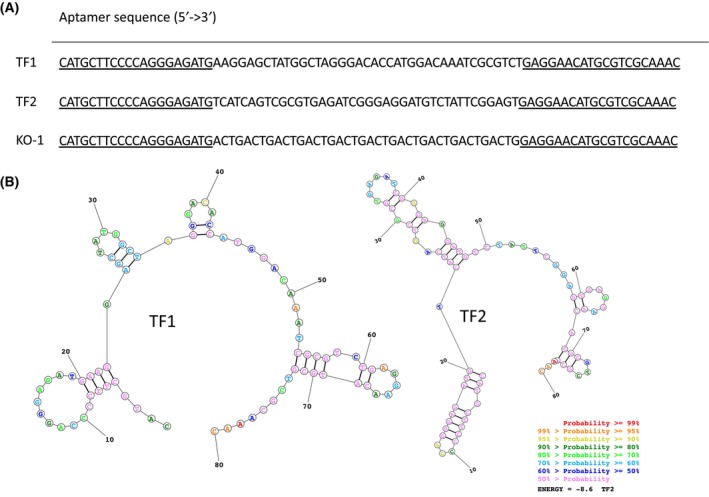
Sequence and structure of aptamers that bind *Tannerella forsythia* cells. (A) Sequences of TF1, TF2 and the negative control KO‐1 aptamers. The fixed‐sequences regions are underlined. (B) Secondary structure prediction using the RNAstructureWeb platform (https://rna.urmc.rochester.edu/RNAstructureWeb/index.html).

Aptamers that bind *T. forsythia* cells were selected using the standard cell‐SELEX method with gradually increasing selective pressure. Two rounds of contra‐selection were included with different bacterial species (*E. coli* and *P. gingivalis*). After eight rounds of selection, the saturation of aptamer binding was achieved. The selected pool of oligonucleotides was sequenced and analyzed. The 10 most abundant aptamer sequences (representing 371 782 sequences in total) formed two clusters based on similarity scores. The two most abundant sequences (TF1 and TF2) represented 7.3% and 7.1% of the entire pool, respectively (Fig. 1A) Secondary structure prediction revealed no obvious similarities (Fig. 1B).

### Analysis of binding affinity

**Fig. 2:**
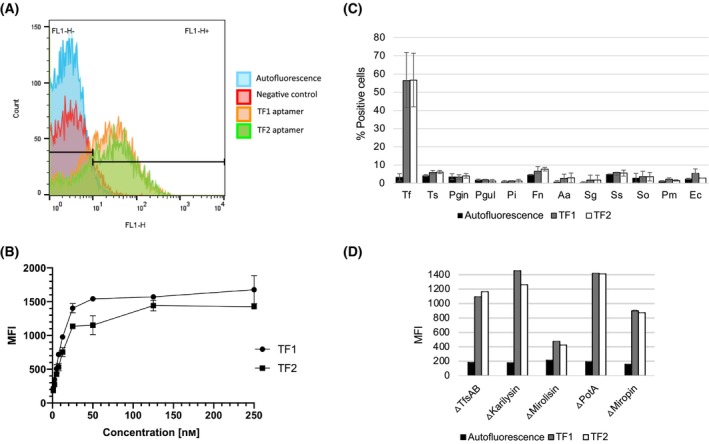
Binding specificity of selected FAM‐labeled aptamers analyzed by flow cytometry. (A) Histogram showing the binding of *Tannerella forsythia* cells to aptamers TF1, TF2 and the negative control aptamer at a concentration of 250 nm. (B) Saturation curve of aptamer binding at concentrations of 0–250 nm in the presence of a constant number of *T. forsythia* cells (6.8 × 10^7^). (C, D) Binding specificity of selected FAM‐labeled aptamers for (C) different bacterial species and (D) different *T. forsythia* knockout mutants. The aptamers were presented at a concentration of 250 nm and for each species or mutant we used 6.8 × 10^7^ bacterial cells. Experiments were carried out twice with two technical replicates. Data are presented as a percent of positive cells or mean fluorescence intensities (MFI) ± standard deviations. Abbreviations used: Aa, *Aggregatibacter actinomycetemcomitans*; Ec, *Escherichia coli*; Fn, *Fusobacterium nucleatum*; Pgin, *Porphyromonas gingivalis*; Pgul, *Porphyromonas gulae*; Pi, *Prevotella intermedia*; Pm, *Parvomonas micra*; Sg, *Streptococcus gordonii*; So, *Streptococcus oralis*; Ss, *Streptococcus sanguinis*; Tf, *Tannerella forsythia*; Ts, *Tannerella serpentiformis*.

The binding of TF1 and TF2 to *T. forsythia* cells was analyzed by flow cytometry, in mixtures containing 250 nm of the aptamer and 6.8 × 10^7^ bacterial cells. Both TF1 and TF2 bound with high affinity to *T. forsythia* cells (Fig. 2A). The highest mean fluorescence intensity (MFI) was observed for TF2, MFI = 63.2 ± 4.1. For TF1 MFI = 55.63 ± 1.72 was measured. The dissociation equilibrium (*K*_d_) was determined by incubating a fixed number of bacteria (6.8 × 10^7^) with different concentrations of the aptamers (0–250 nm) for 1 h before measuring the MFI. We observed typical saturation binding curves and estimates based on nonlinear regression curve fitting yielded *K*_d_ values of 9.86 nm for TF1 and 13.33 nm for TF2 (Fig. 2B).

### Analysis of binding specificity

The specificity of the aptamers was tested by incubating them with various bacteria: *E. coli*, *T. serpentiformis* and the nine oral pathogens: *P. gingivalis*, *P. gulae*, *Pr. intermedia*, *A. actinomycetemcomitans*, *F. nucleatum*, *S. oralis*, *S. sanguinis*, *S. gordonii*, and *Par. micra*. As above, the binding of TF1 and TF2 was analyzed by flow cytometry using 250 nm of each aptamer and 6.8 × 10^7^ bacterial cells. This revealed no statistically significant binding for the bacteria apart from *T. forsythia* cells (Fig. 2C). We also tested a number of *T. forsythia* mutants in the background of the ATCC 43037 strain to identify the binding partner for each aptamer. *T. forsythia* ∆TfsAB mutants, lacking the highly organized semi‐crystalline S‐layer, were still able to bind both aptamers, indicating that neither of the aptamers targeted TfsA or TfsB (Fig. 2D). We also tested mutants deficient for the secreted KLIKK proteases karilysin and mirolysin, and the two cell‐surface protease inhibitors miropin and potempin A [19, 23]. Interestingly, the mirolysin‐deficient mutant showed a significant decrease in binding affinity for both aptamers, with the MFI falling to 11.35% for TF1 and 7.9% for TF2 compared to the wild‐type strain (Fig. 2D). These data indicate that, despite their dissimilar predicted secondary structures, both TF1 and TF2 bind specifically to mirolysin or mirolysin‐modified protein.

## Discussion

Diagnostic aptamers have been developed mainly for the detection of highly pathogenic bacteria that cause acute disease states or represent a serious threat to public health, such as *Mycobacterium tuberculosis*, *Staphylococcus aureus* and *Salmonella enterica* [24]. Periodontal disease is a more challenging diagnostic target because the oral microbiome in humans contains up to 700 bacterial species and its composition varies according to a combination of geographical and socio‐economic factors [25]. Several pathogenic species, known as orange and red complex bacteria, can form microbial consortia with emerging periodontal pathogens. Detailed analysis of the oral microbiome at different stages of periodontal disease reveals that beta diversity initially increases, peaking at the gingivitis stage, followed by a decline during the advanced stages of periodontitis [26]. The contribution of *P. gingivalis*, *T. forsythia* and *Fu. nucleatum* is widely accepted. Although those species are linked to the dysbiotic community, none of them can be regarded as solely responsible for disease initiation and progression. Similarly, some species are found in the healthy periodontium (e.g., *S. sanguinis* or *Rothia aeria*) but can also be found in affected patients [27]. The presence of *P. gingivalis*, *T. forsythia* and *F. alocis* in the saliva provides useful biomarkers correlating with the severity of periodontitis [28].

The bacterial load in periodontitis can be determined by conventional culture‐based methods, immunological detection using an enzyme‐linked immunosorbent assay, quantitative real‐time PCR (mainly targeting *P. gingivalis*), or next‐generation sequencing. However, these methods require specialized equipment and skilled personnel, making them unsuitable for routine diagnostics [12]. This limitation can be overcome by using aptamers for the detection of specific periodontal bacteria. An initial trial reported the development of aptamers for *P. gingivalis*, *Tr. denticola*, and *Streptococcus mutans* [29]. Later, one of the *P. gingivalis* targeting aptamers was used to develop a nanographene oxide theranostic system for antimicrobial photodynamic therapy [30]. In the presented paper, we have expanded the panel of available aptamers by selecting two that bind *T. forsythia* cells with high affinity and specificity. Interestingly, both aptamers appear to bind mirolysin (or a bacteria surface target modified by mirolysin), an associated protease that is secreted via the T9SS. The occurrence of a gene encoding mirolysin limited to *T. forsythia* corroborates with the low cross‐reactivity of the aptamers against other periodontal pathogens, including *T. serpentiformis*. This makes them promising leads for the development of new research tools, diagnostic probes, and even novel therapies for the treatment of periodontitis. However, this requires additional research like optimization of detection approaches tested on multispecies bacterial biofilms and eventually on dental plaque samples. Such a study was beyond the scope of this paper but will be the subject of our follow‐up research aimed to incorporate the presented aptamers into aptasensors setups.

## Conflict of interest

The authors declare no conflict of interest.

### Peer review

The peer review history for this article is available at https://www.webofscience.com/api/gateway/wos/peer‐review/10.1002/2211‐5463.13772.

## Author contributions

DM conceived and designed the project, performed and analyzed experiments, SM analyzed data, AG and BC‐S performed Flow cytometry, DM and JP wrote the manuscript.

## Data Availability

The data that support the findings of this study are available from the corresponding author (danuta.mizgalska@uj.edu.pl) upon reasonable request.
